# Copy-the-majority of instances or individuals? Two approaches to the majority and their consequences for conformist decision-making

**DOI:** 10.1371/journal.pone.0210748

**Published:** 2019-01-25

**Authors:** Thomas J. H. Morgan, Alberto Acerbi, Edwin J. C. van Leeuwen

**Affiliations:** 1 School of Human Evolution and Social Change, Arizona State University, Tempe, Arizona, United States of America; 2 Institute of Human Origins, Arizona State University, Tempe, Arizona, United States of America; 3 Eindhoven University of Technology, Eindhoven, The Netherlands; 4 University of St Andrews, Westburn Lane, St Andrews, Scotland; 5 Max Planck Institute for Psycholinguistics, Wundtlaan 1, Nijmegen, The Netherlands; 6 Centre for Research and Conservation, Royal Zoological Society of Antwerp, Antwerp, Belgium; Middlesex University, UNITED KINGDOM

## Abstract

Cultural evolution is the product of the psychological mechanisms that underlie individual decision making. One commonly studied learning mechanism is a disproportionate preference for majority opinions, known as conformist transmission. While most theoretical and experimental work approaches the majority in terms of the number of individuals that perform a behaviour or hold a belief, some recent experimental studies approach the majority in terms of the number of instances a behaviour is performed. Here, we use a mathematical model to show that disagreement between these two notions of the majority can arise when behavioural variants are performed at different rates, with different salience or in different contexts (variant overrepresentation) and when a subset of the population act as demonstrators to the whole population (model biases). We also show that because conformist transmission changes the distribution of behaviours in a population, how observers approach the majority can cause populations to diverge, and that this can happen even when the two approaches to the majority agree with regards to which behaviour is in the majority. We discuss these results in light of existing findings, ranging from political extremism on twitter to studies of animal foraging behaviour. We conclude that the factors we considered (variant overrepresentation and model biases) are plausibly widespread. As such, it is important to understand how individuals approach the majority in order to understand the effects of majority influence in cultural evolution.

## Introduction

Effective decision making relies on the ability of individuals to combine multiple sources of information in such a way that fitness is maximized. Other individuals can serve as sources of “social” information and patterns in the use of social information have been referred to as “social learning strategies” [1] and “transmission biases” [2]. One social learning strategy of particular interest is “copy the majority” [1] in which observers adopt behaviours performed by the majority of the population. Provided that each individual performs above chance (i.e. individuals are more likely to pick the right option than a wrong one) and that individuals’ decisions are independent, copy-the-majority strategies are highly effective [3] and should be preferred over any copy-the-minority strategy [4].

Conformist transmission [2] describes a subset of copy-the-majority rules, requiring that the probability an individual adopts the majority choice must exceed the proportion of individuals that are in the majority. Thus, if the majority contains 70% of the group’s individuals, conformist transmission implies a greater than 70% chance that a naïve learner would join the majority. Conformist transmission is of particular interest to questions of cultural change as it tends to homogenize groups and stabilise inter-group differences despite differences at the individual level, produced by innovations, copying errors, and migration between groups [5]. Conformist transmission has robust theoretical support as an adaptive learning strategy, with evolutionary models suggesting that it is favoured by selection across the majority of conditions under which selection favours cultural transmission more generally [2,5] (though see [6]). The evidence for conformist transmission in humans was, historically, mixed [7–9] however more recent publications have consistently found evidence in its favour [10–12]. In one such case [10], adult participants were asked to complete a series of mental rotation tasks, and after making each decision participants were shown the decisions of their group mates and given the opportunity to revise their decision. Statistically controlling for the effects of exposure to the task revealed a pattern of social influence consistent with conformist transmission, particularly when the number of group mates was high (i.e. >10).

Whether members of other species engage in conformist transmission is less clear [13]. While evidence for majority biased social learning more generally has been found in many species, strong evidence for conformist transmission specifically comes only from a study of free ranging great tits [14]. In this case, multiple sub-populations were provided with puzzle boxes that could be solved in two different ways and were seeded with demonstrators trained to perform one of the two options. Subsequent observation of untrained birds showed they had a disproportionate tendency to adopt the majority behaviour in their sub-population, consistent with conformist transmission. Despite these findings, studies in fish [15] and fruit flies [16] have produced less conclusive evidence. As such, while conformist transmission seems to be a part of human social learning, its prevalence in other species remains uncertain.

Recently, a discussion has emerged regarding whether a majority is identified in terms of the number of individuals performing a behaviour, or the number of instances in which a behaviour is performed (see [17–20]). Historically, most work on this topic has taken the majority to be defined in terms of the number of individuals, as is the case in studies of group pressure [21,22] or decision-making accuracy [3,5,23]. As a result, most previous work on the copy-the-majority rule, conformity and conformist transmission has used the number of individuals to determine the majority (e.g., [2,5,10,15,17,21,22,24–26]). However, in some cases the majority has been operationalized in terms of the number of *instances* in which each behaviour is performed [14,19,27,28]. In other words, the majority strategy would be the strategy that is observed most often, regardless of how many individuals are responsible for this particular frequency. For example, if in a group of five individuals, two perform behaviour A 25 times each while the other three perform behaviour B 10 times each, behaviour A would be considered the majority strategy. Such an operationalization is not unreasonable. For instance, while much early work describes the majority in terms of individuals, theoretical and experimental designs rarely have the number of instances set against the number of individuals as in the hypothetical example above (though see [29]). As such, it is not clear whether individuals (across different species) respond to the majority of individuals, the majority of instances, or some combination of the two.

Despite this ambiguity, it is plausible that whether individuals attend to instances or individuals can have an effect on cultural evolutionary dynamics when observers engage in conformist transmission. This is because conformist transmission tends to drive which ever behaviour is perceived to be in the majority to fixation. As such, in cases where the two approaches to the majority disagree (i.e. the behaviour performed by the majority of individuals is not the behaviour performed most often), which behaviour spreads may depend on the whether the observers attend to individuals or instances. In what follows we use a mathematical model to assess the conditions under which such disagreement will arise and when it will lead to divergence at the population level.

We consider two factors. The first is “variant overrepresentation”; the tendency of one behaviour to be observed more often than another. Such overrepresentation may arise because one behaviour is performed more frequently, is more salient to observers and so more likely to be noticed or is disproportionately performed in the presence of other individuals. We then consider the effect of model biases where observers attend only to a subset of the population (i.e., based on kin, expertise, dominance, etc., see [30]) and vary the absolute size of the subset of the population that act as demonstrators in addition to the extent to which one behaviour is overrepresented. In all cases, we quantify the likelihood that the majority behaviour, defined in terms of individuals, will differ from that defined in terms of instances.

Our aim is not to provide evidence in favour of one particular conceptualization of majorities (i.e. whether individuals or any given species should attend to individuals or instances), or to provide evidence about which conceptualization individuals of different species use, but rather to identify conditions under which they disagree and/or lead to population level divergence. To contextualize the models, we then discuss empirical cases from a range of natural settings that suggest the factors considered by our models are plausible and may be widespread. We conclude that a diverse array of scenarios can produce a divergence between the majority of individuals and the majority of instances, and consequently recommend further study across multiple species of how observers respond to individuals and instances.

## Models and results

### Variant overrepresentation

Consider a single, local population where each individual performs one of two behaviours, labelled behaviour *A* and behaviour *B*. Let us refer to the proportion of the population performing behaviour *A* as *p*. Therefore, proportion (1-*p*) of the population perform behaviour *B*. Assume individuals who perform behaviour *A* are observed to perform *A* at rate *r* relative to the rate at which individuals who perform behaviour *B* are observed to perform *B*.

For an observer who can see the entire population and who attends to individuals, the perceived frequency of behaviour *A* in the population is *p*. However, for an observer who attends to instances the perceived frequency (*p*_*i*_) is:

$$p_{i}=\frac{pr}{pr+(1-p)}$$ (1)

**Fig 1:**
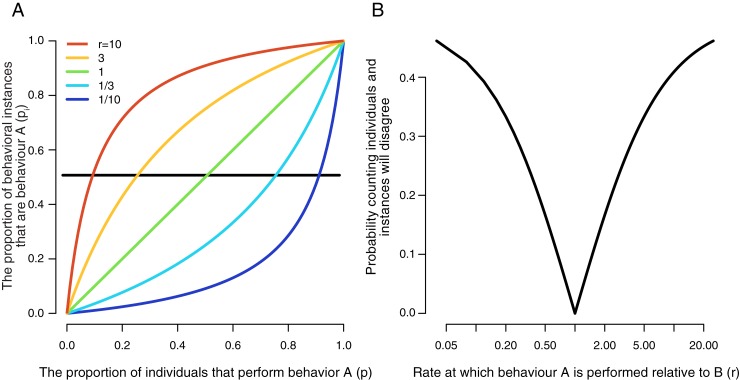
(A) As the proportion of individuals that perform behaviour A (*p*) increases so does the proportion of instances of a behaviour being performed that are behaviour A (*p*_*i*_). However, the rate of increase is strongly affected by the rate at which each individual performs behaviours A or B (*r*). When behaviour A is performed more frequently than behaviour B (*r* > 1) *p*_*i*_ increases rapidly when *p* is low, but this soon slows down as *p* reaches moderately high values because *p*_*i*_ approaches 1. When A is performed less frequently than B (*r* < 1) the increase in *p*_*i*_ is slow at first, but once *p* reaches moderately high values it speeds up dramatically. (B) The probability that two observers who count individuals and instances, respectively, will disagree over which behaviour is in the majority is minimized when *r* = 1, but as *r* increases or decreases, the probability rapidly increases, slowing down as it reaches a maximum value of 1. Note the x-axis is logarithmic to make the symmetry around *r* = 1 more apparent.

The relationship between *p*_*i*_, *p* and *r* is shown in Fig 1A. While increasing *p* always increases *p*_*i*_, *r* modifies the rate at which this occurs. When *r* is 1 the relationship between *p* and *p*_*i*_ is linear and the two are always equal. When *r* < 1, however, increasing *p* has relatively little effect on *p*_*i*_ until high values of *p* are reached, at which point *p*_*i*_ increases dramatically. This causes an observer who counts instances to underestimate the popularity of behaviour *A* relative to an observer who counts individuals. When *r* > 1 the reverse is true; when *p* is low, small increases in *p* cause dramatic increases in *p*_*i*_, but as *p* increases further it has less impact upon *p*_*i*_. This causes an observer who counts *individuals* to underestimate the popularity of behaviour *A* relative to an observer who counts *instances*.

How can we interpret *r*? Perhaps the most direct interpretation is that it describes the rate at which the two behaviours are performed, i.e. *r* = 2 implies that individuals that perform behaviour A do so twice as often as individuals that perform behaviour B (and that all performances are observed). However, alternative interpretations are possible. For instance, *r* can be considered as the salience of the behaviours, i.e. behaviour *A* might be twice as attention grabbing as behaviour *B* and so each instance of its performance is twice as likely to be observed. Another alternative is that *r* is the relative *public* performance rate of the behaviours, i.e. behaviour *B* might be rarely performed in public and so observed instances of its performance could be rare. While we think these alternative interpretations are valid, it is important to note that they are potentially much harder to quantify in an experimental context. For instance, provided the experimenter can continually observe all individuals, counting the number of times each behaviour is performed (the ‘rate’ interpretation of *r*) is quite straightforward. However, even with these data, it may be hard to determine how many of these demonstrations were salient enough to be noted by nearby observers (the ‘salience’ interpretation) or the extent to which different behavioural variants are performed in public versus private (the ‘public’ interpretation). As such, although all three interpretations are consistent with the models, they may be difficult to implement in practice. Nonetheless, we feel it remains instructive to consider cases where all three may be relevant and so we draw on them all in the literature review below.

Any discrepancy between *p* and *p*_*i*_ can lead to differences in the subsequent behaviour of conformist observers depending on whether they attend to individuals or instances. Specifically, a conformist observer who counts individuals will favour option *A* when *p* > 0.5, while an observer who counts instances will favour option *A* when *p*_*i*_ > 0.5. Thus, disagreement between the observers will arise when *p* and *p*_*i*_ are on opposite sides of 0.5, which can be formalized in the following inequality:

$$(p-0.5)(p_{i}-0.5)\leq 0$$ (2)

By inserting Eq (1) into Eq (2) we can produce the following quadratic inequality:

$$p^{2}(2r+2)+p(-r-3)+1\leq 0$$ (3)

This can then be solved to find the boundary points of the inequality which are:

$$p=\frac{1}{2}$$ (4)

and

$$p=\frac{1}{1+r}$$ (5)

Thus, for any value of *r* (other than 1) there is a range of values of *p* for which this inequality is true. Investigation of Fig 1 clearly shows that the values of *p* for which the inequality in Eq (2) is true lie *within* the bounds specified by Eqs (4) and (5)—in terms of Fig 1, it is the region of the x-axis (*p*) for which the y-axis value (*p*_*i*_) and the straight line produced when *r* = 1 are on opposite sides of the horizontal black line (*p*_*i*_ = 0.5). As an example, when *r* = 10 (red line) this region extends from *p* = 1/11 to *p* = 1/2. Assuming that nothing is known of the popularity of the two behaviours (i.e. every value of *p* is equally likely) the size of this region can be considered to be the probability that two observers who count individuals and instances, respectively, will disagree with regards to which behaviour is in the majority.

Eq (5) implies that, as *r* diverges from 1, the probability of disagreement increases quite rapidly, before eventually asymptoting at 0.5 (Fig 1B). At the population level, if *p* lies within Eqs (4) and (5) populations of conformists would converge on different behaviours depending on whether their members counted instances or individuals [2,5]. As an example, consider a case where 26% of individuals perform behaviour A (i.e., *p* = 0.26), but they do so three times as often/saliently as those who perform B (i.e., *r* = 3), leading A to make up 51% of instances. If the population counts individuals, then behaviour B will spread, but if the population counts instances, then behaviour A will spread.

**Fig 2:**
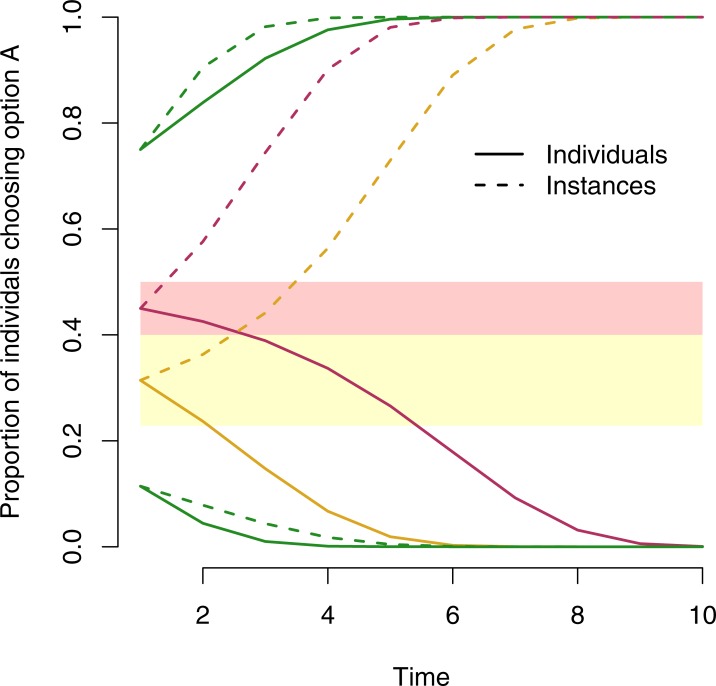
Depending on the starting composition of the population, the two approaches to the majority can either lead to the populations converging on the same behaviour (green lines) or diverging. In the latter case this can be because the two approaches produce disagreement over which option is in the majority (red lines) or because even though the two approaches agree over which option is in the majority they nonetheless disagree sufficiently over the size of this majority (yellow lines). In the case shown *r* = 1.5 and *s* = 1.5 and the lower boundary of the yellow region is 8/35.

This is illustrated in Fig 2, which shows the cultural evolution of populations of conformists who attend to either individuals (solid lines) or instances (dashed lines) starting from four different values of *p*. We follow Morgan *et al*. [31] in using the following form to describe conformist transmission:

$$q=\frac{p^{s}}{p^{s}+{\left(1-p\right)}^{s}}$$ (6)

where *q* is the probability of adopting *A* and *s* is the parameter controlling the strength of conformist transmission (see [11], chapter 2, for a more detailed treatment of this equation). In Fig 2 we consider the case where *s* = *r* = 1.5, i.e. a moderate difference in the rate of performance of the two behaviours, as well as a modest conformist bias.

Note that the populations starting within the region of disagreement (the red region, defined by Eqs 4 and 5) diverge contingent upon whether they attend to instances or individuals. Note also a second region (shaded yellow) which is outside of the region of disagreement (so observers would agree as to which behaviour is in the majority regardless of whether they attend to instances or individuals) but that nonetheless still produces population-level divergence. This is because in this region, although all observers agree as to which behaviour is in the majority, they disagree over the size of the majority to such an extent that they end up diverging. The size of this region is conditional on both the value of *r*, but also on the strength of the observers’ conformist bias and increasing this strength decreases the size of this region (at infinite strength this region disappears entirely).

In the case shown in Fig 2 (where *s* = *r* = 1.5), if a population starts with a value of *p* of 0.31, observers who count individuals will identify that 0.31 of the population have chosen *A*, and according to their conformist bias (Eq 6), they will adopt this behaviour with probability 0.23. Once all members of the population have updated their decision, 0.23 of the population will perform behaviour *A* (i.e., *p* = 0.23) and over subsequent time steps this value will decrease to 0. However, if the population counted instances, again starting from *p* = 0.31, because *r* = 1.5 their estimate of the proportional size of the majority is 0.4 (Eq 1). Due to their conformist bias, they will then adopt *A* with probability 0.36, meaning that although the observers concluded that option *A* was in the minority, its popularity nonetheless increases from its initial value of 0.31, to 0.36. As observers repeatedly update their decision, this increase will continue until *A* entirely dominates the population (see Fig 2).

As such, whether observers count instances or individuals can lead to diametrically opposed outcomes even in cases where observers who count instances or individuals would agree over which behaviour is in the majority. Given this, it may be of interest to know the values of *p* for which this occurs (contingent on *r* and *s*). One bound will always be that specified in Eq 5, for this determines the point at which divergence in cultural evolutionary outcomes would become divergence opinion over which option is in the majority for our pair of hypothetical observers. The other boundary is given by the following equation:

$$p=\frac{{\left(pr\right)}^{s}}{{\left(1-p\right)}^{s}+{\left(pr\right)}^{s}}$$ (7)

We are unable use this equation to give a general solution for *p* as a function of *r* and *s*, nonetheless the insertion of particular values for *r* and *s* does allow the numerical calculation of the corresponding value of *p*, as was done in Fig 2.

### Model biases

Consider now the same population (proportion *p* of which perform behaviour *A*), but assuming a finite subset of *n* individuals act as demonstrators to the entire population. These demonstrators are the same for all individuals in the population and their identity does not change over time. Let us also assume that all learners attend to individuals (not instances). The frequency of behaviour A among the demonstrators (*p*_*d*_) is:

$$p_{d}=\frac{B(n,p)}{n}$$ (8)

where *B(*,*)* is the binomial distribution i.e., the frequency of behaviour A among the demonstrators is the proportion of successes among *n* samples from a binomial distribution with probability of success *p*. Given this, the *expected* value of *p*_*d*_ is *p*, however, for any given value of *n*, *p*_*d*_ will very likely differ from *p*.

We have already established that our two hypothetical observers will disagree over which behaviour is in the majority if *p*_*d*_ falls between the bounds specified by Eqs (4) and (5). As such, with a finite number of demonstrators, the probability of disagreement, given *p*, *r* and *n*, is the probability that *p*_*d*_ falls within these bounds. This can be calculated as the difference between the probabilities that (*i*) *p*_*d*_ is less than or equal to the lower bound and (*ii*) less than or equal to the upper bound:

$$p(disagreement)={|B}^{\prime }\left(\frac{n}{2},n,p\right)-B^{\prime }\left(\frac{n}{1+r},n,p\right)|$$ (9)

where *B’* is the binomial cumulative density function.

**Fig 3:**
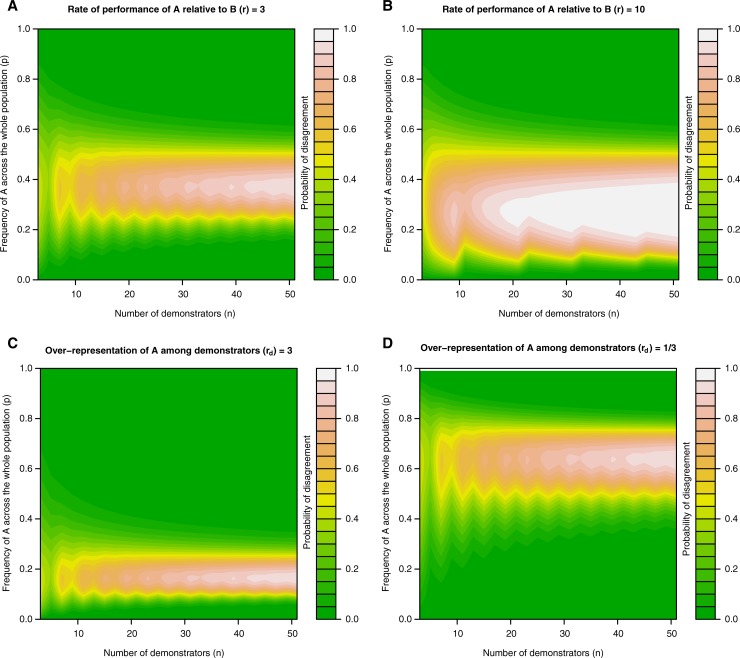
The effect of the number of demonstrators (*n*), the frequency of behaviour A (*p*), the rate of performance of A relative to B (*r*) and the overrepresentation of A among demonstrators (*r*_*d*_) on the probability that two observers who counted individuals and instances, respectively, would disagree over which behaviour is in the majority. (A-B) Certain values of *p* are associated with a raised probability of disagreement. The range of this region increases as r diverges from 1. As *n* increases, the probability of disagreement within this region approaches 1, while, outside of it, it approaches 0. For lower values of *n* the probability is more diffuse, being lower within the region, but higher outside of it. (C-D) Introducing a systematic bias into the demonstrators shifts the values of p that are associated with a high probability of disagreement (*r* is set to 3 in both panels), but the effect of *n* remains the same as in panels A-B.

This equation implies that as *r* diverges from 1, certain combinations of *p* and *n* become increasingly likely to result in disagreement (see Fig 3A and 3B). For high values of *n* this region is that bounded by Eqs (4) and (5)—i.e. with infinite demonstrators this model converges on model 1. For low *n*, the probability of disagreement is lower within these bounds, but greater outside of them—i.e. the chance nature of which individuals are demonstrators makes both agreement and disagreement possible for a wider range of values of *p*.

As before this disagreement has the potential to affect evolutionary dynamics. Assuming that the identity of the demonstrators remains steady over time (i.e. they are not re-sampled for every individual, but are a stable feature of the population, for instance being copied due to their age, status or success, c.f. [32]), then when disagreement would arise, a population of conformists who copy individuals would converge on different behaviours than a population of conformists who count instances.

In the above equations we assume that the behaviour performed by an individual does not affect the probability that they are a demonstrator. However, in many instances (see below) this is not the case. As such, we now consider a case where there is a systematic bias in the demonstrators such that individuals who perform behaviour *A* are *r*_*d*_ times more likely to be a demonstrator than individuals who perform behaviour *B*. The probability of disagreement between an observer who counts instances and an observer who counts individuals remains the probability that *p*_*d*_ falls within the range specified by Eqs (4) and (5) and can be calculated as:

$$p(disagreement)=\left|B^{\prime }(\frac{n}{2},n,\frac{{pr}_{d}}{pr_{d}+1-p})-B^{\prime }(\frac{n}{1+r},n,\frac{{pr}_{d}}{pr_{d}+1-p})\right|$$ (10)

This systematic bias among demonstrators shifts the region of high disagreement to different values of *p*, but does not qualitatively change its structure (see Fig 3C and 3D). As a note we are here only considering the probability of disagreement between the two observers and not (*i*) the probability of population level divergence (i.e. we are not considering the region where the observers agree, but divergence nonetheless occurs), or (*ii*) the probability that a single observer would reach a different judgement when learning from the demonstrators compared to the same observer given access to the entire population. Nonetheless, it is likely that systematic biases among the demonstrators greatly increase the likelihood of the demonstrators being un-representative of the population as a whole.

### Summary

The models presented above highlight that when one behaviour is performed more frequently, saliently or publicly than another (variant overrepresentation), observers can come to different conclusions with regards to which behaviour is in the majority, depending on whether they count individuals or instances. This discrepancy becomes more pronounced as the relative frequency/salience/public-ness of the two behaviours diverge. Moreover, this discrepancy has population level consequences: whenever counting instances or individuals would lead to disagreement, the approach used determines which behaviour will spread under conformist transmission. This can occur even when the two approaches to the majority agree, with a conformist response to counting instances causing the apparent minority behaviour to spread through the population conditional on the strength of the observers’ conformist bias.

We also considered the likelihood of disagreement between the two approaches to the majority in the context of the observers having access to only a subset of the population (model biases). We find that the stochasticity of sampling a finite number of individuals increases the range of conditions under which disagreement can arise, although it also decreases the probability of disagreement in areas where it was previously guaranteed. Allowing an individual’s behaviour to affect the probability they are a demonstrator changes which frequencies of *A* (i.e. values of *p*) are likely to produce disagreement, but does not otherwise change our results.

## Empirical cases of variant overrepresentation and model biases

So far, we have demonstrated that counts of individuals and instances may, in theory, be in opposition to each other when one behaviour is performed more frequently, more saliently, or more publicly than another. We now review published accounts in order to assess the plausibility of these circumstances across species, with a special focus on scenarios in which the least common variant (in terms of individuals) is the most common (in terms of instances). Our goal is to provide evidence for the plausibility of variant overrepresentation and model biases in a wide range of contexts, without making any specific claims about precisely how widespread these phenomena are.

### The ‘rate’ interpretation of r: One behaviour is performed more than the other(s)

In this category, we include cases in which behaviours are performed at different rates. One human case illustrating this point is a recent study of Twitter activity. Shore et al. [33], studying a large sample of 2,7 million users, quantified the “degree of extremism” by analysing the political orientation of the webpages shared by users. They found that the apparent prevalence of extremist views is in fact due to a minority core of highly active users, who happen to link to more extremist sources. Counting instances would therefore lead to the conclusion that Behaviour A (“sharing moderate political views”) is less common than behaviour B (“sharing extremist political views”), but counting across how many individuals these instances were distributed would lead to the opposite conclusion.

A relevant non-human case concerns social learning dynamics in wild great tits. Aplin et al. [14] provided a task with two possible solutions to populations of wild birds. In groups seeded with demonstrator birds trained on a particular solution, the seeded solution was the majority of instances and was also performed by the majority of individuals. However, in one of the three control conditions (where the task was introduced without trained demonstrators), the solution performed by the majority of *individuals* was not the same as the behaviour performed most often (Aplin et al., 2015, supplementary information). In other words, a discrepancy between individuals and instances appeared endogenously within this population (with an estimated *r* value of 1.2).

### The ‘salience’ and ‘public’ interpretations of r

Above, we noted that the *r* parameter can also be interpreted as the relative ‘salience’ or ‘public-ness’ of the two behaviours. While these interpretations have limitations in terms of how easy they are to document experimentally, we will nonetheless describe known cases that appear to reflect such a disparity.

We will first consider the ‘salience’ interpretation of *r*. Here we include scenarios in which one of the behavioural variants is more noticeable and, as a consequence, observed more reliably, than the other. For instance, cognitive anthropologists (e.g., [34,35]) have hypothesised that cultural dynamics are influenced by general biases toward perceiving and adopting certain kinds of information. One such case in the context of painted art is the spread of direct gaze in portraiture (a style in which the subject looks straight towards the observer), which has been argued to be due to the fact that such portraits are more memorable and attention-catching that those in which the subject does looks elsewhere [36]. As such, if we label indirect gaze as behaviour *B*, this corresponds to *r* > 1.

Many other traits have been argued to be culturally successful due to their cognitive “attractiveness” [37]. For instance, stories that are emotionally laden [38] or heavy with threats [39] are much better retained along diffusion chains than equally frequently uttered stories lacking these high-arousal elements. Given that such stories are more memorable, when an observer aggregates their memories to determine the prevalence of different stories, they are likely to inflate the estimate of their prevalence.

Another scenario in which relative salience may bias response-outcomes pertains to experimental puzzle-solving. In “two-action method” studies (in which subjects of various species must choose between two actions to solve a puzzle) it is often the case that one of the two actions is spontaneously preferred by the subjects, which could be due to increased salience of the action to observers. For instance, chimpanzees showed a general preference for a “poking” strategy over a “lifting” strategy, likely because “poking” more closely resembles natural foraging behaviours and so could draw observers’ attention [25,40]. Thus, even if a demonstrator performed the lifting strategy in view of observers, they may not register its performance due to a lack of salience.

We will now consider cases of relevance to the ‘public’ interpretation of *r*: even if two behaviours are performed at the same rate, and with the same salience, one can be far less visible to observers if it is typically performed in private. One classic example of a behaviour being disproportionately performed in the presence of others is alcohol use in young individuals, especially at university campuses [41,42]. Among campus students, behaviour *A* (drinking, over-drinking, and expressing enjoyment for it) is generally, if not exclusively, performed in public situations, while behaviour *B* (expressing regret for drinking) is not expressed, or expressed predominantly in private situations. In addition, drinking alcoholic beverages may also be more salient–memorable, attention-catching–than drinking non-alcoholic ones (see our previous point). Centola et al. [42] reported other examples of behaviours that tend to be over-represented because they are publicly performed, including support for vegetarianism, homophobia, or the purchasing of luxury goods like costly automobiles, high-end fashion clothes, or expensive wine.

An analogous dynamic has been noticed for medical treatments. Studying reviews of cholesterol and weight loss treatments on the website amazon.com, de Barra [43] reported that "people who have a positive outcome tend to tell more people about their disease/treatment than people with poor or average outcomes”. If information A is “cholesterol treatment X is good” and information B is “cholesterol treatment X is bad”, even if the majority of individuals did not experience positive effects, information A will be more likely to be shared, and may appear to be more common.

The opposite effect relates to behaviours that are performed generally when alone, and hence will tend to be under-estimated. Behaviours considered deviant in a particular society, such as drug usage, homosexuality, or visiting prostitutes, could belong to this category [44]. In this case, behaviour *A* (e.g. drug usage) is under-represented in the public arena, giving rise to the suggestion that drug usage might be less common than it actually is.

### One individual is observed more than others

In model 2, a subset of individuals served as demonstrators to the whole population, and we explored how this affects the probability of disagreement between observers using the two definitions of the majority (in terms of instances or individuals). Such uneven connectivity is practically universal for real human social networks which are, from hunter-gatherer societies [45] to contemporary mobile communications [46], characterized by the presence of “fat tails” (i.e. few nodes with many connections, and many nodes with few connections). One potential outcome of this is that the traits of well-connected individuals can appear to be in the majority even if an observer using the same notion of the majority, but with access to the full population, would disagree (i.e. it increases the probability that the demonstrators are not representative of the population as a whole). This has been referred to as the “majority illusion” [47] and has been documented in networks with various structures, including scale-free networks.

Similar network structures exist in numerous non-human animal species [48–50]. For instance, networks in chimpanzees, great tits, and whales (based on e.g., co-presence) are non-randomly structured [51–53]. This means that among non-human animals certain individuals are likely to be disproportionately influential and members of these groups could also be prone to the “majority illusion”.

We also modelled a case where the probability an individual is a demonstrator is affected by their behaviour. Many empirical cases suggest that this can occur. Any attribute that is correlated with network degree will tend to be overrepresented amongst the socially influential [54]. For example, if a social network is built based on friendship connections [45], highly central individuals will have, by definition, more friends than less central individuals. If an observer would “copy” the typical number of friends, she will (by definition) observe central individuals more often, who in turn will have more friends than average, leading to the observer ending up with a biased estimate. In support of this, it has been found that people tend to believe their social lives are less rich than the social lives of others. This is likely related to the fact that famous and highly connected personalities (“socialites”) are over-sampled when assessing typical sociability [55]. In general, if any “behaviour A” is somehow linked to “having many friends”, for example cooperative attitudes, but also, say, risk propensity, this will also be over-estimated when counting instances of behaviours as compared to counting instances of individuals.

Other less obvious attributes could be correlated with network centrality. Apicella et al. [45] showed that several features, including height, age, body fat, and marital status, are also correlated with network degree. If, for example, innovators tend to have a more central position in social networks, behaviour-based sampling will favour the spreading of innovations (where individual-based sampling may result in a more conservative trend); on the contrary, if central individuals are also more conservative, innovations will tend to be under-estimated. Future studies are needed to explore in depth the relation between individual features and network position in human [56] and non-human animals [57].

Similar patterns have been observed in non-human species. For instance, recent studies increasingly indicate that animal personalities (operationalized in terms of temporal consistency in the speed by which novel challenges are explored, labelled: “boldness”) are both predictive of network centrality, and thus visibility for others [58], and preferred foraging tactics [59–63]. For instance, bold rooks consistently use different food handling tactics than shy rooks, implying that, based on the plausibility of increased saliency of bold rooks (in terms of being first to act, being sole actor versus one of the group, and engaging more frequently due to lack of fear), observers may obtain a distorted record of the distribution of tactics across group members [59].

## Conclusion

The work presented here uses a modelling approach to demonstrate that different observers who identify the majority behaviour by counting instances or by counting individuals, respectively, may disagree over which behaviour is in the majority when there is variation in the rate at which different behavioural variants are performed. Moreover, we show that having a subset of the population acting as demonstrators to the population as a whole, changes the probability of such disagreement occurring. Finally, as above, we note that existing theory [5] makes clear that in such cases, populations composed of conformist learners who count instances or individuals, respectively, will converge on different behaviours and so differences at the level of majority identification can have population level consequences.

These findings emphasize the importance of understanding whether observers attend to individuals, instances, or perhaps more likely, combine the two, in studies of conformist social learning [64]. In many laboratory experiments, where the experimenter has control over the social information provided to observers, this issue has been avoided by ensuring each demonstrator individual is only observed for a single instance [10]. However, in more naturalistic settings such controls are typically not available and so analysing the data in terms of instances or individuals is an important choice on the behalf of the researcher. There is no reason, *a priori*, to favour one over the other. Nonetheless, where experimental designs allow the potential for disagreement between the number of individuals and instances, we encourage further work exploring if and how observers of different species use this information.

Although the literature cited above discusses many cases where there is the potential for disagreement between counts of instances and individuals, this will not always be the case. When *r* is close to 1, for instance, the window within which disagreement will arise is narrow (although if conformist transmission is weak the window for divergence-without-disagreement may be larger). Moreover, once conformist transmission acts to homogenise the population and the popular behaviour spreads, then counts of instances and individuals will come into agreement even if they previously disagreed. As such we should not necessarily expect to see such disagreement in the case of well-established traditions, although such disagreement may well have influenced which behaviours became well-established in the first place. Finally, it is important to note that even if there is a disagreement between instances and individuals at the level of the overall population, this may not be apparent to observers if the population is spatially structure and observers only sample a subset of the population. This is plausibly the case with the great tit data discussed above [14] where disagreement existed across one control population, but not within the sub-populations associated with the three feeders therein (D. Farine pers. comm.).

Putting this specific case aside, the literature reviewed above suggests that disagreements between the number of individuals and instances could plausibly arise at the level at which observers collect data in a range of species and contexts. As such, the difference between individuals and instances is likely to affect the cultural evolutionary dynamics, as well as the success of individual decisions under either learning rule. Future work could conduct evolutionary analyses of the two strategies to identify conditions under which either is favoured over the other. For instance, a highly conformist strategy that counts individuals fares poorly when the environment changes, as it hinders the spread of new information [6] (though see [65] for a case where conformist transmission combined with asocial learning can accommodate environmental change). However, whether the same is true of a conformist strategy that counts instances remains unclear. There is also scope for further empirical work to determine whether different species use (or are even capable of) one or the other of these strategies. One reason to expect that observers might attend to the number of individuals is that, while the behaviour of different individuals may or may not be independent, multiple instances of behaviour from a single individual are clearly not. As such, an observer attending to the number of instances may be susceptible to pseudoreplication. In keeping with this, data suggest that humans, and perhaps chimpanzees, are sensitive to violations of independence across multiple observations and take this into account when combining information [29,66]. Nonetheless, this may well be different in other species. In particular, tracking individuals may be a cognitively demanding task beyond the abilities of many species. Alternatively, in cases of “public information use” [67,68] the frequency with which the behaviour is performed contains valuable information (for instance feeding behaviour frequency may indicate the availability of food) and so attending to instances can be a valuable approach.

Cultural evolution is the product of the decisions made by a group of individuals. As such, the details of the cultural evolutionary process rest upon the psychological mechanisms possessed by individuals. Here, we have argued that what had previously been treated as a single behaviour—conformist transmission and/or “copy-the-majority” bias—can and should be considered in light of what observers are enumerating: individuals or instances. These different behaviours can be empirically distinguished and produce different cultural evolutionary dynamics under a range of plausible conditions.
